# A novel method for coastal zone bathymetry based on multisensor data fusion and unmanned systems

**DOI:** 10.1038/s41598-025-19550-1

**Published:** 2025-10-13

**Authors:** Oktawia Specht, Andrzej Stateczny

**Affiliations:** 1 Department of Transport, Gdynia Maritime University, Morska 81-87, 81-225 Gdynia, Poland; 2 Department of Autonomous Systems, Gdynia Maritime University, Morska 81-87, 81-225 Gdynia, Poland

**Keywords:** Bathymetric monitoring, Coastal zone, Data fusion, Geospatial data, Multisensor integration, Engineering, Ocean sciences, Solid Earth sciences

## Abstract

Existing geospatial data fusion methods in hydrography do not take into account the accuracy of individual measurements when creating a bathymetric map. Consequently, geospatial data acquired by devices with low depth measurement accuracy may lead to a deterioration in the accuracy of coastal zone topography. To address this limitation, this study presents a novel method for coastal bathymetric monitoring based on the integration of multimodal geospatial data collected by unmanned platforms equipped with on-board sensors. These include Single-Beam Echo Sounder (SBES) and MultiBeam EchoSounder (MBES), a photogrammetric camera, and Light Detection and Ranging (LiDAR) from Airborne Laser Scanning (ALS) and Mobile Laser Scanning (MLS). As part of this method, bathymetric and photogrammetric data are processed using three modules: processing depth data, processing shallow-water data, and determining the coastline. After processing, the data are fused using an original weighted average data fusion method, in which weights for individual data sources are determined based on the measurement accuracy. The results demonstrate that the proposed coastal monitoring method effectively minimises redundant geospatial inputs. Notably, the model is parametric, and its accuracy depends on the appropriate selection of processing parameters and fusion settings.

## Introduction

Bathymetric monitoring involves investigating underwater terrain in lakes, rivers, seas, and oceans to generate detailed bathymetric maps. The resolution and reliability of these charts are closely tied to the quality, precision, and spatial distribution of collected depth data, which can vary depending on the measurement technologies used. Inaccuracies or inconsistencies in bathymetric mapping can lead to incorrect identification of navigation conditions, placement of anchorages, or estimation of safe operational depths in ports and waterways^1,2^.

A bathymetric map is a visual representation of seafloor topography, typically using detailed depth contours (isobaths) or colour-coded surfaces to depict the size, shape, and distribution of underwater features. Such maps are widely used in marine research, engineering, and environmental analysis. In contrast, a bathymetric model is a digital representation of seafloor depth values arranged in a grid, where horizontal and vertical lines intersect to form squares, each containing a depth value. Commonly used in topographic mapping, these models serve as the analytical foundation for creating bathymetric maps and are essential for data fusion, numerical modelling, and quantitative analysis^1,2^.

To address these challenges, various measurement systems have been developed to enhance the precision and efficiency of depth data acquisition. Hydroacoustic instruments, typically including devices such as a Single-Beam Echo Sounder (SBES), a Multibeam EchoSounder (MBES), a Global Navigation Satellite System (GNSS), an Inertial Navigation System (INS), and a Sound Velocity Profiler (SVP), are widely used for collecting bathymetric measurements and increasingly being mounted on Unmanned Surface Vehicles (USVs)^3,4^. Additionally, optoelectronic devices such as Light Detection and Ranging (LiDAR), photogrammetric cameras, GNSS, and INS are frequently installed on Unmanned Aerial Vehicles (UAVs)^5^. Shoreline measurement, especially along the 0 m isobath, is carried out using high-precision geodetic tools like Terrestrial Laser Scanners (TLS), total stations, or GNSS-based systems. Moreover, photogrammetric cameras and LiDAR, which are mounted on UAVs, are increasingly being utilised for this purpose. Furthermore, the process of shoreline determination based on LiDAR or photogrammetric camera data is supported by various algorithms designed for the automatic detection of the shoreline trajectory.

Advanced bathymetric data acquisition techniques enable the collection of a large number of measurement points while ensuring high (often nearly 100%) seabed coverage with bathymetric measurements. Consequently, the resulting data can meet the highest precision criteria applicable to hydrographic surveys, namely, Exclusive Order, Special Order and Order 1a, as outlined in *the S-44 Standards for Hydrographic Surveys* issued by the International Hydrographic Organization (IHO)^6^. However, it should be noted that geospatial data acquired using various on-board sensors of unmanned measurement platforms differ in depth measurement accuracy, which significantly affects the quality of the developed bathymetric maps.

**Fig. 1:**
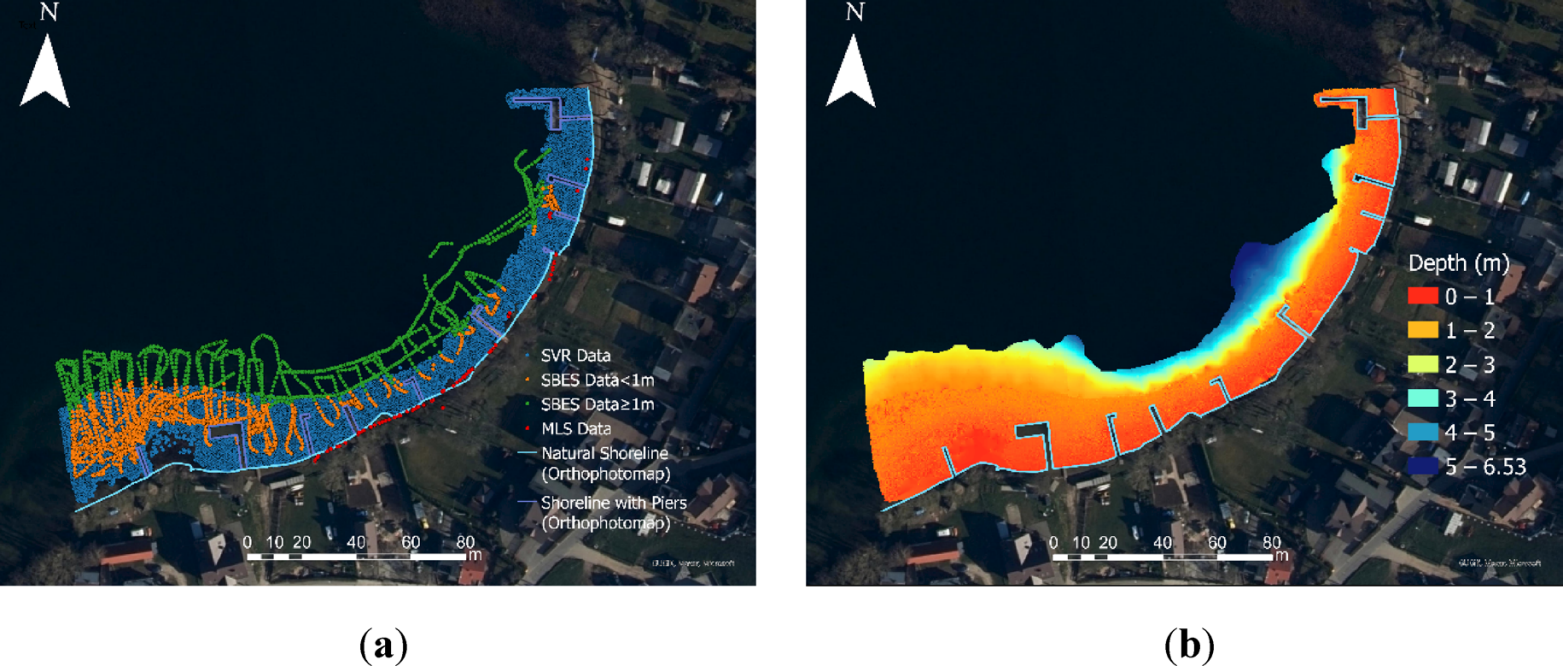
Spatial distribution of geospatial data acquired via on-board sensors of unmanned platforms (**a**) and corresponding bathymetric representation of the study area (**b**) in UTM/WGS 84 zone 34N (EPSG: 32634).

As previously mentioned, measurement points recorded using on-board sensors of measurement platforms are characterised by varying depth measurement accuracy and result in data redundancy in certain areas. Consequently, hydrographers face a time-consuming and labour-intensive process of bathymetric data processing, aimed at accurately and faithfully representing the topography of the coastal zone. For instance, geospatial data in the study area were acquired using methods and devices such as Support Vector Regression (SVR), SBES, Mobile Laser Scanning (MLS), and UAVs. It should be noted that the SVR method is used to determine depths in areas where direct measurements with a GNSS Real-Time Kinematic (RTK) receiver cannot be obtained. This is achieved by using a Structure-from-motion (SfM) point cloud in combination with depth measurements collected with a geodetic receiver. Figure 1 presents the spatial distribution of geospatial data captured by on-board sensors mounted on unmanned platforms, alongside the resulting bathymetric map for the study site.

To ensure the generation of a reliable bathymetric map, it was necessary to minimise the number of depth measurement points obtained from various sensor systems mounted on unmanned measurement platforms. The effective use of multimodal geospatial data poses challenges related to data processing, analysis, and integration. To properly execute all stages, a novel approach for coastal zone bathymetry is proposed. This methodology supports the creation of bathymetric representations with enhanced accuracy, optimised for the detailed mapping of the coastal environment.

Current geospatial data fusion methods in hydrography, including methods such as Nearest Neighbour, Natural Neighbour Interpolation (NNI), Inverse Distance Weighting (IDW), or geostatistical interpolation, do not account for the measurement accuracy of individual measurement devices during the bathymetric map development process. As a result, geospatial data acquired by devices with low depth accuracy may lead to a deterioration in the representation accuracy of the shoreline topography.

Existing methods of geospatial data fusion use interpolated values, which emphasise the need for new solutions. Considering navigation safety and the accuracy of the bathymetric map, it is essential to develop a new method that preserves the actual measured data values (position and depth). The necessity of representing depths on a bathymetric map remains regardless of the chosen scale, while ensuring the required fidelity of surface representation, maintaining position accuracy, and preserving minimum depth values crucial for the safety of navigation.

This article proposes a novel approach to coastal zone bathymetric monitoring by integrating multimodal geospatial datasets acquired through on-board sensor systems installed on unmanned measurement platforms. The proposed method was developed through a detailed analysis of a representative dataset. The availability of high-quality multimodal geospatial data enabled a comprehensive assessment of current processing techniques and their limitations. Drawing on these insights and practical experience, a coherent framework for bathymetric monitoring using multisensor data fusion was established. Consequently, the complete description of the proposed monitoring method is presented in the Results section, reflecting the final integrated outcome of the study. In contrast, the Methodology section focuses on the processing steps applied to each dataset, which collectively informed the structure of the final method.

## Results

### Stages of the coastal zone bathymetric monitoring method

**Fig. 2:**
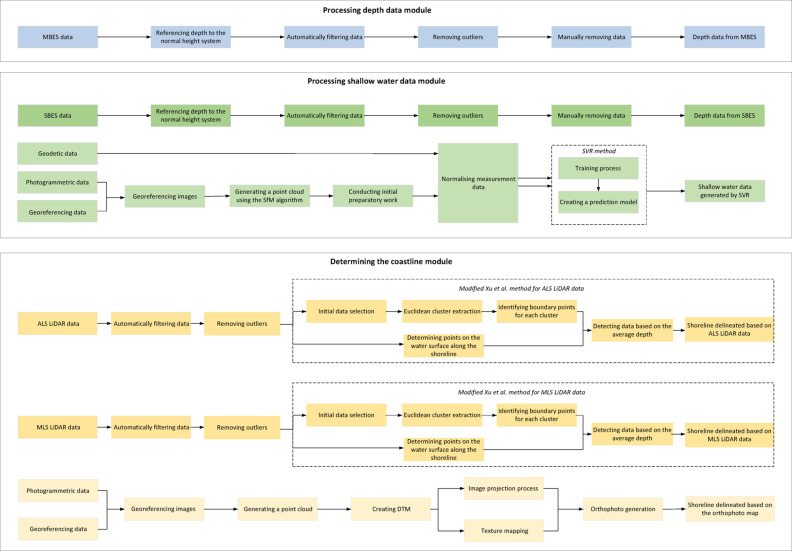
Modules for processing measurement data.

Within the coastal zone bathymetric monitoring method, geospatial data are processed using three modules: processing depth data, processing shallow-water data, and determining the coastline (Fig. 2). Additionally, a novel weighted average data fusion method will be described, in which the weights assigned to individual data sources are determined based on measurement accuracy.

**Fig. 3:**
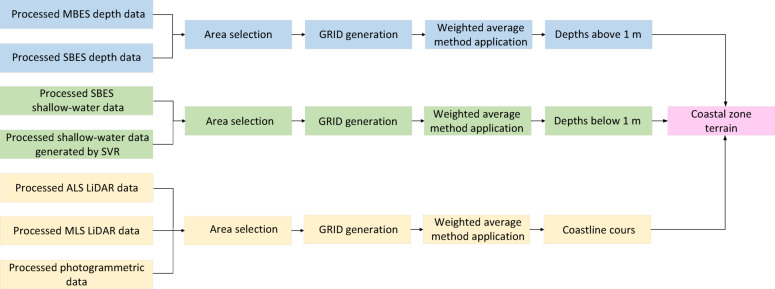
Multisensor data fusion module.

The final module of the coastal zone bathymetric monitoring method is multisensor data fusion (Fig. 3). Within this module, data acquired from on-board sensors of unmanned survey platforms will be integrated to reconstruct coastal zone terrain.

To apply the weighted average method, it is first necessary to determine the weights for each dataset. For this purpose, the formula used to compute the weighting function in the IDW approach was modified. Initially, this method assigns weights based on the spatial distance between an observed point and the corresponding interpolated value^7^. In the context of multisensor data fusion, this distance parameter was replaced with the measurement accuracy of a given point. As a result, the weighting function in the weighted average method can be expressed as follows:

1 $$w_{i} = \frac{1}{{\left( {a_{i} } \right)^{u} }}$$

where *w*_*i*_—weight of the *i*-th point, *a*_*i*_—measurement accuracy of depth/planar rectangular coordinates of the *i*-th point (*p* = 0.95), *u*—power exponent.

As an illustrative example, if the depth data acquired using an SBES system have a measurement accuracy of 0.06 m, the corresponding weight can be calculated using Eq. (1) with a power exponent of *u* = 1, as follows:

2 $$w = \frac{1}{{\left( {0.06} \right)^{1} }} = 16.67.$$

For comparison, using *u* = 2:

3 $$w = \frac{1}{{\left( {0.06} \right)^{2} }} = 277.78.$$

It should be noted that weighting coefficients decrease as the measurement accuracy of a given point decreases. The second factor influencing the determination of a point’s depth is the power exponent, which is commonly used in the Inverse Distance to a Power (IDP) method^8,9^. Based on studies in this field, the power exponent value most commonly adopted for computing the weighting function is either 1 or 2^10,11^.

With the weighting function determined, the weighted average depth in the *m*-th cell of the regular square grid is obtained according to the formula below:

4 $$\overline{d}_{m} = \frac{{\sum\nolimits_{i}^{M} {w_{i} \cdot d_{i} } }}{{\sum\nolimits_{i}^{M} {w_{i} } }} = \frac{{\sum\nolimits_{i}^{M} {\frac{{d_{i} }}{{\left( {a_{i} } \right)^{u} }}} }}{{\sum\nolimits_{i}^{M} {\frac{1}{{\left( {a_{i} } \right)^{u} }}} }}$$

where $\overline{d}_{m}$—weighted average depth in the *m*-th cell of the grid, *M*—number of points located within the *m*-th cell of the grid, *d*_*i*_—depth of the *i*-th point.

The calculation of the weighted average depth can be illustrated using a single grid cell containing three depth values: two acquired using the SBES system (0.12 m and 0.11 m), and one obtained from the SVR method (0.17 m). The SBES measurements are characterised by a high measurement accuracy of 0.06 m, which corresponds to a weight of 16.67 for a power exponent *u* = 1. The SVR-derived point has a lower accuracy of 0.23 m, resulting in a weight of 4.35. Using Eq. (4), the weighted average depth for this cell is calculated as:

5 $$\overline{d}_{m} = \frac{(0.12 \cdot 16.67) + (0.11 \cdot 16.67) + (0.17 \cdot 4.35)}{{16.67 + 16.67 + 4.35}} = 0.1213\;{\text{m}}{.}$$

As stated in Eq. (4), the weighted average depth method cannot be applied to shoreline points, as they always have the same depth (0 m). For this reason, the weighted average method for planar rectangular coordinates was adopted for these points. This method assumes that, initially, grid cells containing shoreline points are selected. Only then can the weighted average of the planar rectangular coordinates be determined within the cells of the regular square grid, as follows:

6 $$\overline{x}_{m} = \frac{{\sum\nolimits_{i}^{M} {w_{i} \cdot x_{i} } }}{{\sum\nolimits_{i}^{M} {w_{i} } }} = \frac{{\sum\nolimits_{i}^{M} {\frac{{x_{i} }}{{\left( {a_{i} } \right)^{u} }}} }}{{\sum\nolimits_{i}^{M} {\frac{1}{{\left( {a_{i} } \right)^{u} }}} }}$$

7 $$\overline{y}_{m} = \frac{{\sum\nolimits_{i}^{M} {w_{i} \cdot y_{i} } }}{{\sum\nolimits_{i}^{M} {w_{i} } }} = \frac{{\sum\nolimits_{i}^{M} {\frac{{y_{i} }}{{\left( {a_{i} } \right)^{u} }}} }}{{\sum\nolimits_{i}^{M} {\frac{1}{{\left( {a_{i} } \right)^{u} }}} }}$$

where $\overline{x}_{m} ,\overline{y}_{m}$—weighted average of planar rectangular coordinates in the *m*-th cell of the grid containing shoreline points.

### Implementation and evaluation of the coastal zone bathymetric monitoring method

**Fig. 4:**
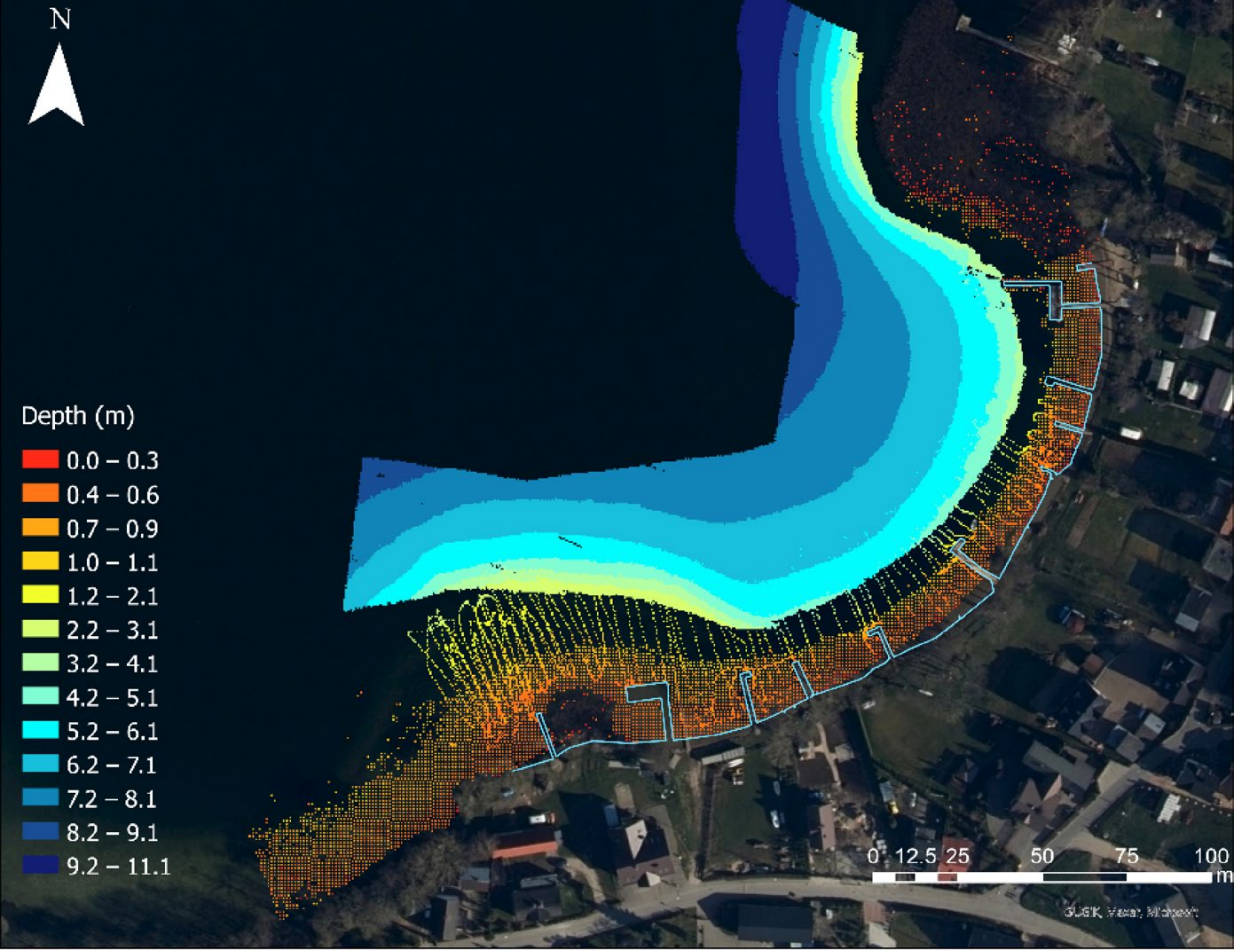
Spatial location of the geospatial dataset collected during field measurements conducted at Lake Kłodno in UTM/WGS 84 zone 34N (EPSG: 32,634).

After cleaning the bathymetric data, the integration process can begin. The first step is to ensure that all datasets are aligned within a consistent planar coordinate system. Additionally, further data cleaning is recommended. With all the data visible, erroneous points can be identified and corrected. This is particularly relevant for data obtained using the SVR method. In the case of SVR data, some points covered a small area with depths exceeding 3 m, which led to their exclusion. Furthermore, SVR data within the shoreline boundaries were removed. This is due to the occurrence of wave refraction near the shoreline, which prevents accurate depth determination of the waterbody. Figure 4 shows the location of the processed geospatial data collected as part of the field survey carried out at Lake Kłodno.

As can be seen in Fig. 4, the natural shoreline (without piers and other constructions) drawn based on the orthophotomap was not included. This was omitted because, for this particular waterbody, a shoreline that consists of the piers is more appropriate. This decision is based on the fact that in the developed coastal zone, the bathymetric monitoring method allows for only one shoreline to be selected.

The next step involves calculating the weights for each dataset. The table summarising the weights for each measurement dataset collected during the depth and image-based data gathered from the Lake Kłodno study site is presented in Table 1.

**Table 1:** Summary dataset weights derived from the geospatial measurements conducted at Lake Kłodno.

Data	Depth/plane coordinate accuracy (*p* = 0.95)	*w* (*u* = 1)	*w* (*u* = 2)
MBES^a^	0.15	6.67	44.44
SBES (depth range 1–7.09 m)^b^	0.06	16.67	277.78
SBES (depth range 0.4–0.99 m)^b^	0.07	14.29	204.08
SVR^b^	0.23	4.35	18.90
ALS LiDAR^b^	3.36	0.30	0.09
MLS LiDAR^b^	2.43	0.41	0.17
Photogrammetric (natural shoreline)^b^	0.07	14.29	204.08
Photogrammetric (shoreline with piers)^b^	0.08	12.50	156.25

^a^Measurement accuracy (*p* = 0.95) determined from device technical specifications.

^b^Measurement accuracy (*p* = 0.95) estimated based on comparison with reference data.

As shown in Table 1, the assigned weight tends to increase with measurement accuracy for the power exponent (*u* = 1). However, increasing the exponent from 1 to 2 resulted in higher weights for datasets with measurement accuracy greater than 1 m (MBES, SBES, SVR, and photogrammetric data), while weights decreased for datasets with accuracy below 1 m (LiDAR).

Subsequently, all bathymetric datasets were merged. A grid layer was generated using 0.5-m resolution cells, as this resolution enables accurate representation of terrain relief. Using a regular square grid with a larger cell size may result in significant errors due to the increased spacing between measurement points.

**Fig. 5:**
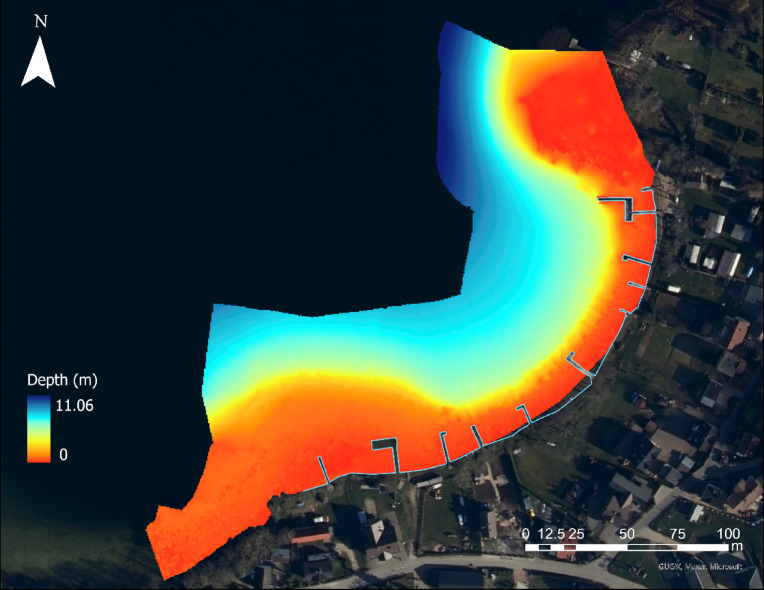
Weighted average data fusion model with a power exponent of 1 in the local height system for Lake Kłodno in UTM/WGS 84 zone 34N (EPSG: 32,634).

To comprehensively represent the study area and depict the topography of the coastal zone, depth values were assigned to empty grid cells using the natural neighbour method^12^. This interpolation technique was selected based on previous studies demonstrating its effectiveness with multimodal datasets. The Natural Neighbour method estimates values for unsampled locations by identifying neighbouring input points using Voronoi cells and assigning weights according to the proportion of overlap between the new point’s cell and those of its neighbours^13^. According to the research results^13^, the natural neighbour method achieved the highest coefficient of determination (0.999). Therefore, the natural neighbour method was applied solely for data visualisation purposes and was not used to evaluate the precision of the weighted average data fusion model. Figure 5 shows the weighted average data fusion model with a power exponent of 1 in the local height system for Lake Kłodno.

**Fig. 6:**
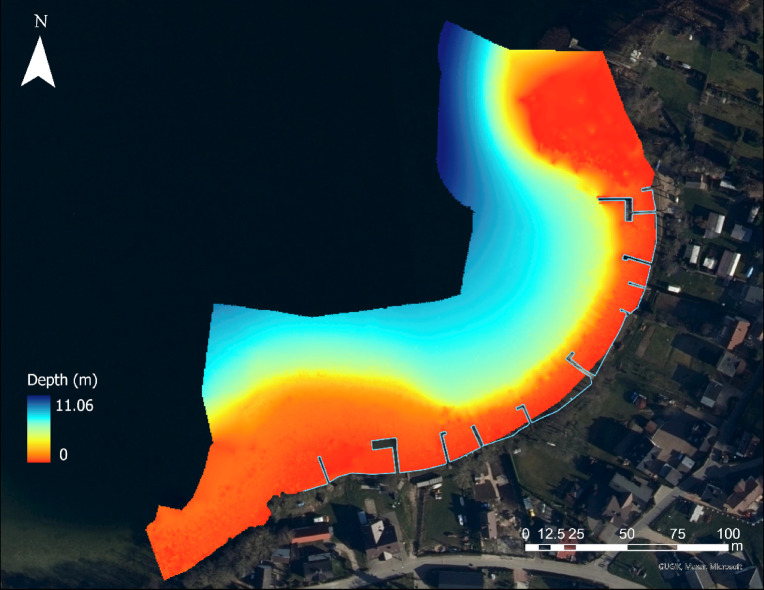
Weighted average data fusion model with an exponent of 2 in the local height system for Lake Kłodno in UTM/WGS 84 zone 34N (EPSG: 32,634).

As is evident in the resulting model (Fig. 5), the shoreline can be distinctly observed, as the grid cells were generated only in areas where bathymetric data were available. The highest density of grid cells occurs in the area surveyed by the MBES echosounder. Conversely, the lowest point density appears within the range of the 1 m and 3 m isobaths, where SBES echosounder data were used. Notably, the measurement data obtained using the SVR method played a significant role. These data enabled extensive coverage in hard-to-reach areas. The maximum and minimum depths of the waterbody are 11.06 m and 0 m, respectively. The average recorded depth is 3.93 m, while the Root Mean Square (RMS) depth is 3.17 m. The proposed weighted average data fusion method achieved an RMSE of 0.393 m and an R^2^ of 0.9997 for both models, confirming its high accuracy and reliability. The proposed fusion model for *u* = 1 and *u* = 2 achieved a Root Mean Square Error (RMSE) of 0.0394 m, with a 95% confidence interval of 0.0393–0.0394 m, and an R^2^ value of 0.9997 with an approximate 95% confidence interval of 0.9656–1.0000. The standard deviation of prediction errors was 0.0394 m, nearly equal to the RMSE, indicating a consistent error magnitude across the validation dataset. These results confirm the high accuracy and robustness of the method across the validation dataset. The quantitative analysis performed did not provide grounds to reject the thesis that the proposed bathymetric monitoring method will enable obtaining the data quality required in accordance with the IHO Special Order requirements. Figure 6 shows the weighted average data fusion model with an exponent of 2 in the local elevation system for Lake Kłodno.

Visually, Figs. 5 and 6 appear identical. This is due to the use of the same depth scale. Furthermore, the maximum and minimum depths of the waterbody are 11.06 m and 0 m, respectively. The mean depth calculated for the waterbody was 3.93 m, while the RMS depth reached 3.17 m. An important observation is that the difference in average depth between the grid cells of the two models was 0.0004 m.

## Discussion

The proposed methodology was evaluated against selected studies from the literature to assess its effectiveness in coastal bathymetric monitoring. Table 2 summarises the key parameters of the presented method in comparison with similar approaches from recent publications.

**Table 2:** Comparison of selected interpolation methods used in bathymetric studies across different waterbodies.

Study	Method	Waterbody	Accuracy [RMSE (m)/R^2^ (–)]
This study	Weighted average data fusion	Lake Kłodno, Poland	0.04 m/0.99
Satpute et al.^14^	IDW, NNI, Kriging	Mauritian coast	IDW: 0.68 m NN: 0.89 m Kriging: 0.98–1.46 m
Genchi et al.^15^	IDW	Argentine coast	Bathymetry: 0.18 m Topography: 0.09 m
Amoroso et al.^16^	IDW, LPI^a^, Kriging (OK), RBF^b^	Gulf of Naples	IDW: 1.43 m/0.993 LPI: 6.41 m/0.858 OK: 0.92 m/0.997 RBF: 1.08 m/0.996
Amante and Eakins^17^	Triangulation	Kachemak Bay, Alaska	Spline: highest accuracy (no RMSE explicitly given)
Lewicka^13^	NNI	Lake Kłodno, Poland	NNI: 0.030 m/0.999

^a^LPI—Local polynomial interpolation.

^b^RBF—Radial basis function interpolation.

With complete confidence, it can be stated that the proposed fusion method achieved the most accurate results compared to previous studies. The results obtained using the proposed weighted average data fusion method demonstrate a high level of accuracy. However, as evidenced by the author’s earlier research, significant efforts had already been made to explore the application of multisensor geospatial data in the development of bathymetric models. Nevertheless, it was the implementation of the author’s fusion method that yielded the most substantial improvement in model accuracy, clearly demonstrating the advantage of integrating complementary data sources through a customised and systematic algorithmic approach.

Despite its high accuracy, the proposed method has certain limitations that should be acknowledged. First, it relies heavily on the availability and quality of multisensor geospatial data, which may not always be accessible in all study areas or under all conditions. The method was tested in a relatively calm, freshwater lake environment, with full sensor availability and favourable measurement conditions. Its applicability in more challenging settings, such as turbid estuaries, rocky coastal zones, or densely vegetated riverbanks, may be limited by factors such as water turbidity, dynamic wave activity, and aquatic vegetation, all of which can introduce noise into the photogrammetric dataset, particularly when applying the SVR method. Moreover, UAV-based measurements depend on stable weather and hydrometeorological conditions, including clear skies, no precipitation, and wind speeds below 6–7 m/s. Deviations from these conditions may impair image quality, reduce positional accuracy, or even prevent successful data collection.

In addition to environmental factors, further limitations may arise during the data preparation and processing stages. These include the misclassification of noise as outliers, the accidental removal of valid measurements, the incorrect weighting of individual data sources, and errors in referencing data to the vertical datum (e.g., sea level). Each of these factors may negatively affect the quality of the resulting bathymetric model if not carefully validated and controlled.

Despite these limitations, the proposed method is designed to be adaptable to different sensor configurations. The fusion strategy dynamically recalculates weights based on the accuracy of the available input data, and the grid resolution can be adjusted accordingly. In cases where specific datasets, such as MBES or LiDAR, are not available, the method can still be applied using alternative sources such as SBES and UAV-derived photogrammetric data. However, users should be aware that omitting high-resolution datasets may result in localised reductions in model accuracy, particularly in complex nearshore areas.

Given these constraints, it is also essential to account for operational requirements. The practical implementation of the proposed method depends on several operational factors, including sensor configuration, data acquisition time, software used, and computational resources. These aspects may vary depending on the available technology and the complexity of the surveyed area. In this study, data acquisition was carried out using multiple unmanned platforms equipped with LiDAR sensors (airborne and mobile) and a multibeam echosounder. Data collection for these components was managed using HYPACK® software, which ensured accurate synchronisation and positioning. Among all recorded datasets, UAV-based photogrammetric imagery required the largest amount of storage space. During a single aerial flight over Lake Kłodno, 376 images were acquired, resulting in a total data volume of approximately 4.48 GB.

Regarding data storage and processing, the total volume depends on the number of sensors used and the spatial extent of the survey. While typical LiDAR and hydroacoustic datasets are manageable on modern workstations, high-resolution photogrammetric processing (e.g., using SfM techniques) may require a system with at least 32 GB of RAM and a dedicated GPU to efficiently generate dense point clouds. Overall processing time may range from several hours to more than a day, depending on the dataset size. Once the raw datasets are processed, the final bathymetric model is developed in a Geographic Information System (GIS) environment. This step involves data integration, interpolation, and visualisation and can typically be completed within one working day, provided that the input datasets have been preprocessed and the system meets minimum performance requirements.

## Conclusions

This article presents the development of a novel approach for generating a digital model of the coastal zone using geospatial data. The method places particular emphasis on collecting accurate depth data with a vertical accuracy reaching 1 m. This task is challenging when using traditional manned hydrographic vessels due to their significant draft. The main objective of the research was to develop a bathymetric monitoring approach dedicated to coastal areas, based on the integration of multimodal geospatial data acquired from on-board sensors installed on unmanned measurement platforms. The data sources include, among others, single-beam and multibeam echo sounders, a photogrammetric imaging system, as well as airborne and terrestrial LiDAR sensors. As part of this method, bathymetric and photogrammetric data are processed using three modules: processing depth data, processing shallow-water data, and determining the coastline.

After processing the geospatial data, they are integrated using the original weighted average data fusion method, where weights for individual data sources are determined based on measurement accuracy. It is worth emphasising that the original method includes a shoreline extraction technique using LiDAR data and an SVR method, which allows for the acquisition of shallow-water depths up to 1 m based on SfM point clouds from UAV data.

Based on the analysis carried out, the following conclusions were drawn:

- The method for bathymetric monitoring of the coastal zone is parametric. Its effectiveness depends on the selection of parameters when processing individual data and creating a fusion model;
- Depth data obtained using the SVR method allowed for extensive data coverage in hard-to-reach places. However, they have the worst depth measurement accuracy of all the sensors used;
- LiDAR data from Airborne Laser Scanning (ALS) and MLS processed using a modified shoreline extraction method that takes into account the presence of terrain obstacles near the boundary between land and water should be supplemented with additional data. Therefore, the method for determining the coastline based on an orthophotomap is considered the main source of data about its course;
- Research has shown that the proposed shoreline extraction method allows for much more accurate determination of its natural course using MLS than ALS;
- The proposed weighted average data fusion method achieved an RMSE of 0.393 m and an R^2^ of 0.9997 for both models, confirming its high accuracy and reliability;
- The modular design of the proposed approach makes it suitable for further development through the environmental adaptation of sensors and their combinations, depending on specific site characteristics and data availability;
- The increase in the power exponent in the bathymetric monitoring method of the coastal zone does not affect the accuracy of the developed weighted average data fusion model.

## Study area and data

**Fig. 7:**
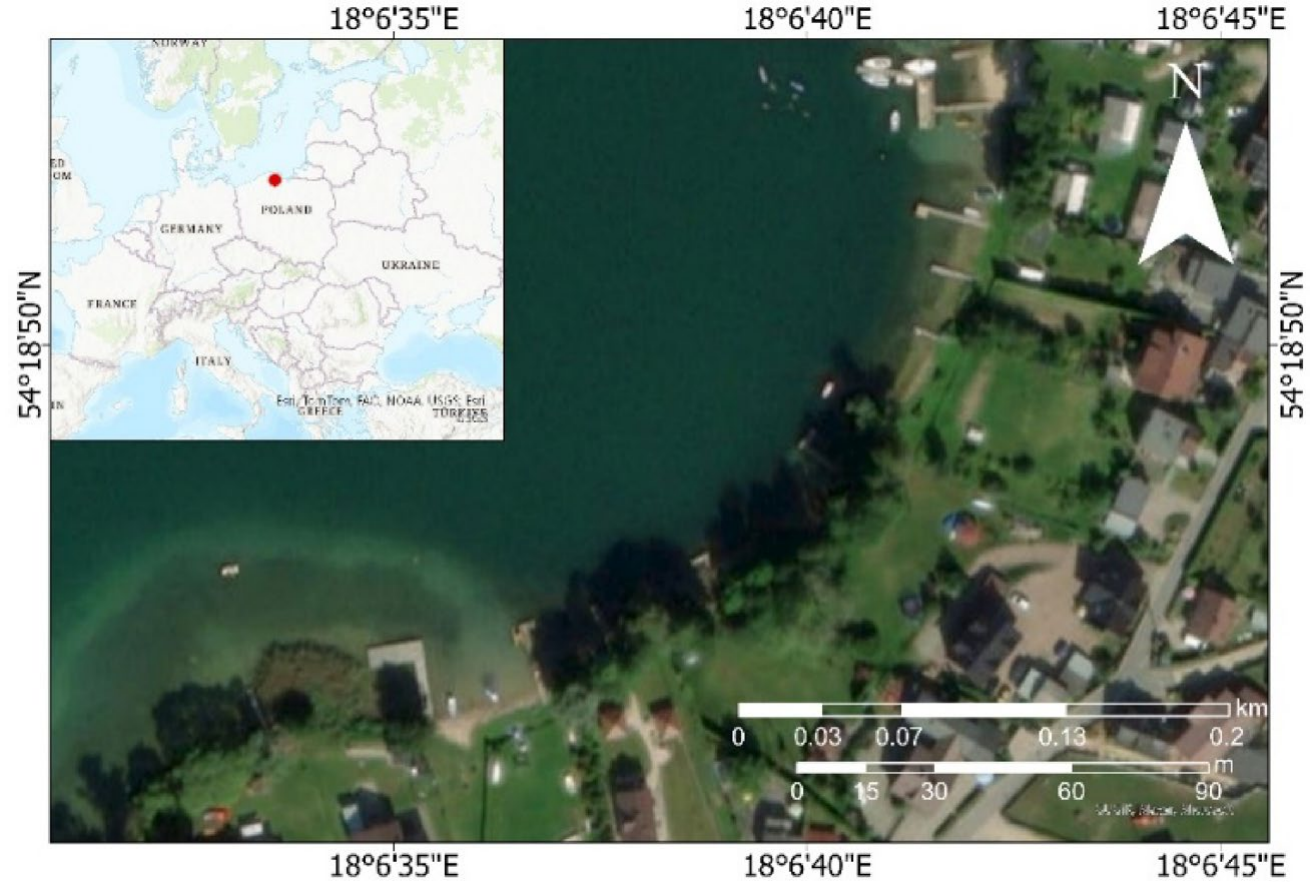
Location of the bathymetric and photogrammetric measurements conducted on Lake Kłodno in WGS84 (EPSG:4326).

To develop a method for bathymetric monitoring of transitional aquatic areas, a decision was made to conduct the study at Lake Kłodno (Poland), which has a moderately balanced ecological condition. The study area is situated along the lake’s shoreline and encompasses both terrestrial and aquatic sections of the reservoir. The shoreline is covered with trees (Fig. 7).

During the measurement campaign conducted on Lake Kłodno, the following data were recorded:

- SBES data acquired with a SonarMite BTX echosounder, coupled with a Trimble R10 GNSS RTK receiver and deployed on the AutoDron USV;
- MBES data collected with a PING 3DSS-DX-450 interferometric sonar system, installed on the HydroDron-1 USV;
- SVR data obtained using a 1-inch, 20 MP Complementary Metal–Oxide–Semiconductor (CMOS) imaging sensor, deployed on the DJI Phantom 4 RTK UAV;
- ALS LiDAR data acquired with a Velodyne Puck VLP-16, integrated with an SBG Ellipse-D GNSS/INS system and mounted on the Aurelia X8 Standard LE UAV;
- MLS LiDAR data acquired with a Velodyne Puck VLP-16, integrated with an SBG Ekinox2-U GNSS/INS system and mounted on the HydroDron-1 USV;
- Photogrammetric data acquired with a 1-inch, 20 MP CMOS sensor integrated into the DJI Phantom 4 RTK UAV.

## Methodology

### Processing SBES and MBES depth data

Among the most widely used instruments are single-beam and multibeam echosounders. The presented approach to processing data from an SBES can also be applied to data recorded using an MBES. Moreover, it assumes that bathymetric measurements are conducted using a USV equipped with either an MBES or SBES, as well as a GNSS RTK receiver.

Bathymetric measurements obtained using SBES or MBES systems are assigned planar rectangular coordinates from differential GNSS RTK positioning, together with the depth values recorded by the hydroacoustic device. The first stage of processing bathymetric data from an MBES or SBES involves referencing the depth measurements to the official vertical reference system of the respective country. In this study, conducted in Poland, all depth values were referenced to the PL-EVRF2007-NH vertical datum, where the reference level (*H* = 0.000 m) corresponds to the Amsterdam Ordnance Datum^18^.

**Fig. 8:**
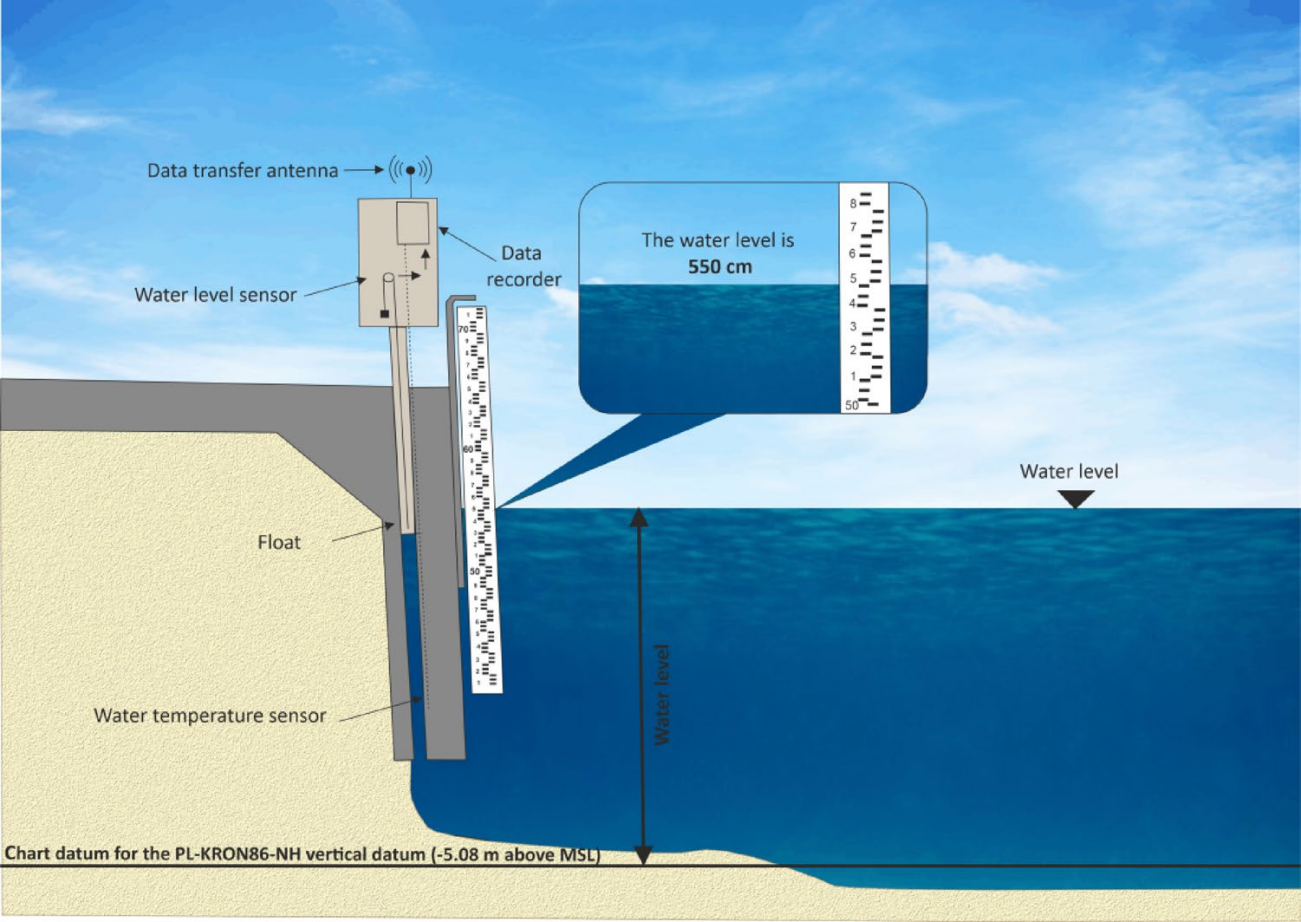
Schematic representation of water level and temperature observations performed by the Institute of Meteorology and Water Management—National Research Institute (IMGW-PIB) in relation to the PL-EVRF2007-NH vertical datum (own elaboration based on^19^).

The method to determine the normal height of the measurement point in the PL-EVRF2007-NH vertical datum was applied according to Lewicka et al.^19^, where the relevant equations are described in detail. This process involves referencing the observed sea level relative to the established chart datum. In Poland, for instance, the Amsterdam height system is used, with the chart datum reference level set at 500 cm^18^. The sea level is averaged based on observations recorded by a tide gauge between consecutive full hours. A crucial factor in determining the depth correction is the appropriate selection of the hydrometeorological station, which should be located as close as possible to the bathymetric measurement site. In the absence of a nearby hydrometric station, the reference water level should be obtained from the closest tide staff, as illustrated in Fig. 8.

If no hydrometeorological station is available in the study area or its vicinity, and there is no wave activity during the bathymetric survey, the measured depths can be referenced to the water level height. In such cases, the water level is determined based on averaged elevation measurements conducted along the 0 m isobath using a GNSS RTK receiver. Alternatively, shoreline detection can be performed using other techniques, such as LiDAR scanning.

The next stage of the process is the removal of erroneously recorded depth values. In the case of MBES and SBES devices, this includes filtering out measurements that are artificially increased due to multipath acoustic reflections, a common issue in shallow-water environments. Any remaining erroneous depths are then reduced from the survey profiles. All remaining erroneous depth values are then removed from the survey profiles. Additional outliers can be excluded either manually or by applying statistical outlier detection techniques^20^.

### Image-based depth estimation using UAV data

Another instrument for acquiring bathymetric data is a photogrammetric camera. Digital imagery from this camera is processed to generate a point cloud using the SfM technique. This point cloud is then used to develop a predictive model, which is subsequently applied to determine depth using the SVR method^21^.

**Fig. 9:**
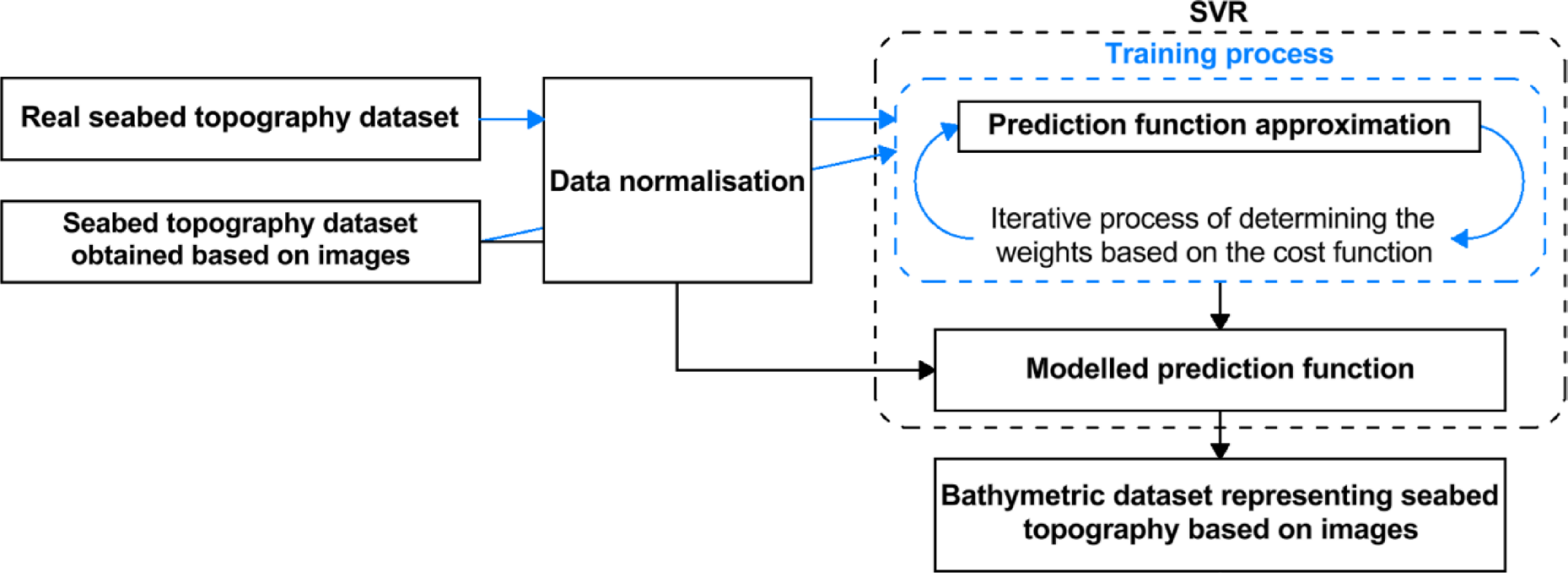
Diagram illustrating the operation of the SVR algorithm according to^24^ (own elaboration based on^19^).

In the context of coastal bathymetric studies, the SVR algorithm^21^ was applied to estimate shallow-water depths using aerial imagery acquired via UAVs. The SVR approach (Fig. 9)^22^ operates by mapping data into a high-dimensional space and fitting a linear regression model^23^. This technique addresses the issue of light refraction at the interface between air and water. Additionally, the model-based representation of the seabed facilitates the automation of the depth estimation workflow. The method is particularly effective when applied under stable weather conditions, such as in windless environments, allowing for reliable photogrammetric processing using UAV-based systems.

Work on the SVR model should begin with preliminary data preparation, which serves as the basis for building the prediction model. The initial step involves filtering the point cloud to exclude points located outside the area of interest, particularly those situated on land. Depth calculations are then referenced to the water surface elevation, measured using a GNSS RTK receiver. To ensure consistency, it is advisable to eliminate all points situated above the water surface, as well as those that exceed the defined maximum depth threshold. According to previous findings^25^, the maximum depth considered in the analysis was 1 m.

In the following step, reference points for survey profiles were generated. These were derived from the SfM-based point cloud by calculating the average depth of the nearest neighbouring points, within ± 10 cm of the GNSS RTK position.

To improve the reliability of the predictive model, the dataset should be filtered to eliminate potential measurement noise. This filtering can be performed manually or by applying the 2*σ* statistical criterion, which involves removing points whose deviation from the mean value exceeds 2*σ* (95.4% of observations). Once the data has been appropriately processed, it is then possible to calculate a new mean depth value for each point.

The final stage of training data preparation is data normalisation. This is a necessary process due to the SVR algorithm’s sensitivity to large-scale differences between individual features of the input data, such as depth^26^. Among various techniques, Z-score normalisation is one of the most frequently applied methods for standardising data^27^.

After assembling the training set, the depth measurements are normalised and used as the basis for constructing a predictive model of the seafloor geometry. In this context, the SVR technique is applied, which aims to generate a linear approximation that retains the highest number of valid samples within an acceptable error threshold *ε*. The goal is to ensure that most of the measured depth values fall within a defined margin from the predicted regression curve. The SVR formulation is expressed in Eq. (8), commonly recognised in the literature as the initial optimisation objective for this method^28^

8 $$\mathop {\min }\limits_{w} f(w),f(w) \equiv \frac{1}{2} \cdot w^{T} \cdot w + C \cdot \sum\limits_{i = 1}^{N} {\xi_{\varepsilon } \left( {w;d_{i} ,d_{{ref_{i} }} } \right)}$$

where *f*(*w*)—approximated prediction function, *w*—vector containing weight coefficient, *C*—hyperparameter defining the impact of the cost function on weight vector optimisation, *N*—total number of samples in the training set, *ε*—hyperparameter defining the maximum deviation, the distance from the approximated regression line, *ξ*_*ε*_—a distance from ε margin for training sample located beyond the predefined deviation (*ξ*_*ε*_ > 0), $d_{{ref_{i} }}$—reference (normalised) depth value.

The complete and detailed methodology for determining shallow-water depths using the SVR algorithm, including data preprocessing, model optimisation, and performance evaluation, is described in^24,25^. In the final phase, the SVR algorithm is implemented as a linear regression model, which is used to estimate shallow-water depths. It is worth emphasising that the correction mechanism shifts the position of points within the point cloud depending on the measured depth. Such correction is significant for submerged points, which are affected by refractive distortion governed by Snell’s law^29^.

### Shoreline extraction using ALS and MLS LiDAR data and orthophotomaps

The first step in processing the raw LiDAR scans involves merging them into a single point cloud. It is important to note that point clouds recorded using GNSS and INS systems are already georeferenced. However, if only LiDAR data were collected, the georeferencing process must be completed before further processing, using ground control points measured with a GNSS RTK receiver^30^.

Before further analysis, the LiDAR point cloud must also be cleaned to remove echoes and noise introduced during data acquisition. There are also traditional methods for LiDAR point cloud denoising, such as local surface fitting using the Moving Least Squares (MLS) method^31^, value averaging, or statistical approaches. Additionally, machine learning techniques are increasingly being used for LiDAR data filtering^32^.

**Fig. 10:**
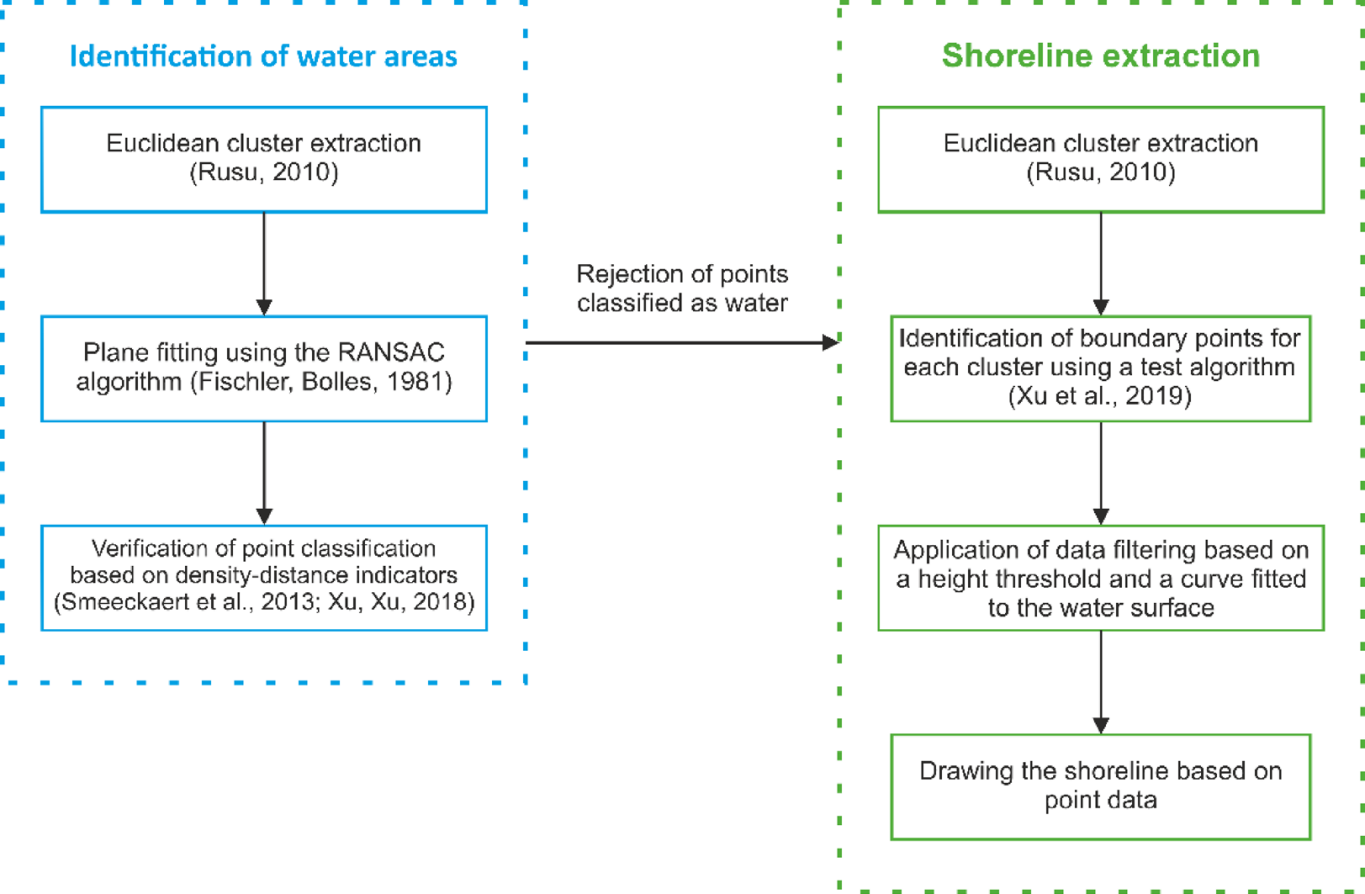
Flowchart presenting the successive steps of the adapted shoreline extraction proposed by Xu et al.^33^ (own elaboration based on^19^).

The parametric method developed by Xu et al.^33^ was ultimately selected because it consistently delivers higher shoreline accuracy (up to 1 m) under variable coastal conditions. Additionally, the choice of this method was motivated by its flexibility in optimising variables to suit specific types of waterbodies, shoreline characteristics, and measurement conditions (Fig. 10).

The initial step in the procedure focuses on detecting and eliminating points that belong to the water surface. To achieve this, points within the LiDAR dataset are clustered using the Euclidean extraction method^34,35^. An alternative parametric approach proposed by Halicki et al. reduces complexity by requiring only the minimum cluster size^36^. The second principal stage of the process applies the RANSAC algorithm to fit planes^37^. The above stages, point clustering and plane fitting, have been described in detail in previous studies^32,35,38,39^ and are therefore only briefly summarised here.

**Fig. 11:**
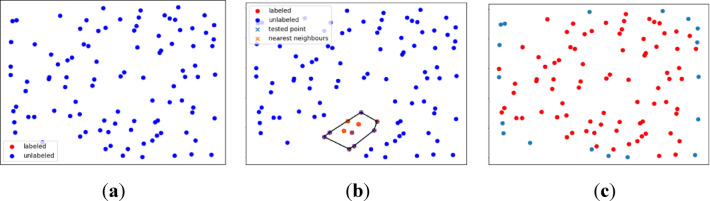
A diagram illustrating the application of the test algorithm^33^ for an artificial dataset of 100 points. The initialisation of the test algorithm on the test dataset (**a**), the first convex hull generated within the test algorithm (**b**), and the final result of the test algorithm (**c**)^36^.

The next step of the work involves selecting boundary points that form the shoreline for a given cluster using a test algorithm^33^. This method assumes that during the initialisation phase, all points are treated as unlabeled. Furthermore, for a given point *P*, a convex hull is created based on its *k*-nearest neighbours. Points located inside the convex hull are then labelled as classified. Additionally, point *P* within the convex hull is excluded from the shoreline set *S*_*w*_ if it is located inside a triangle formed by three vertices belonging to *S*_*w*_^40^. This procedure is repeated until all possible points are classified. The remaining unlabelled points in the final stage represent the boundary points forming the shoreline (Fig. 11). Furthermore, the algorithm allows for the removal of points that are located farther than *T*_*d*_ from the remaining points.

After determining the boundary points, the authors of the modified shoreline extraction method proposed data filtering based on the so-called elevation threshold. The authors of the modified shoreline extraction method set the elevation threshold at 2.5 m above the water surface level for inland waterbody^36^.

The next filtering process is based on a curve determined on the water, running along the shoreline. The first step is to define a curve consisting of *N*_*w*_ user-drawn points, located along the shoreline at a relatively constant distance from the shore. Next, it is necessary to calculate the mean Euclidean distance from the created curve to all potential boundary points. The second step involves filtering based on the mean distance from the generated curve. Points that are farther than the mean distance should be removed. The final step requires the user to manually determine the shoreline based on the remaining points.

An orthophotomap serves as an important reference for identifying shoreline boundaries, enabling the precise determination of the boundary between land and water in satellite or aerial images. This process can be carried out using manual digitisation or Machine Learning (ML) techniques. The ML approach involves training models on large datasets of shoreline imagery, allowing the model to learn and recognise distinctive shoreline features and patterns. Therefore, the development of an effective model requires access to extensive datasets that include orthophotomaps with clearly visible shorelines^41,42^.

## Data Availability

The datasets generated and analysed during the current study are not publicly available due to institutional data sharing policies, but are available from the corresponding author on reasonable request.
